# *In Vitro* Assembly and Stabilization of Dengue and Zika Virus Envelope Protein Homo-Dimers

**DOI:** 10.1038/s41598-017-04767-6

**Published:** 2017-07-03

**Authors:** Stefan W. Metz, Emily N. Gallichotte, Alex Brackbill, Lakshmanane Premkumar, Michael J. Miley, Ralph Baric, Aravinda M. de Silva

**Affiliations:** 1 Department of Microbiology and Immunology, University of North Carolina School of Medicine, Chapel Hill, NC USA; 2 Department of Pharmacology, University of North Carolina School of Medicine, Chapel Hill, NC USA; 3 Department of Epidemiology, University of North Carolina School of Public Health, Chapel Hill, NC USA

## Abstract

Zika virus (ZIKV) and the 4 dengue virus (DENV) serotypes are mosquito-borne *Flaviviruses* that are associated with severe neuronal and hemorrhagic syndromes. The mature flavivirus infectious virion has 90 envelope (E) protein homo-dimers that pack tightly to form a smooth protein coat with icosahedral symmetry. Human antibodies that strongly neutralize ZIKV and DENVs recognize complex quaternary structure epitopes displayed on E-homo-dimers and higher order structures. The ZIKV and DENV E protein expressed as a soluble protein is mainly a monomer that does not display quaternary epitopes, which may explain the modest success with soluble recombinant E (sRecE) as a vaccine and diagnostic antigen. New strategies are needed to design recombinant immunogens that display these critical immune targets. Here we present two novel methods for building or stabilizing *in vitro* E-protein homo-dimers that display quaternary epitopes. In the first approach we immobilize sRecE to enable subsequent dimer generation. As an alternate method, we describe the use of human mAbs to stabilize homo-dimers in solution. The ability to produce recombinant E protein dimers displaying quaternary structure epitopes is an important advance with applications in flavivirus diagnostics and vaccine development.

## Introduction

Zika virus (ZIKV) and the dengue viruses (DENVs) are mosquito-borne members of the *flaviviridea* family, which can cause severe neurological and hemorrhagic syndromes in humans^1–3^. It is estimated that 400 million DENV infections occur each year and that over half of the world’s population live in countries with active DENV or ZIKV transmission^3^.

The continuing threat of DENVs and, more recently, ZIKV has stimulated much work on different vaccine platforms including live attenuated virus, inactivated whole virus, protein subunit and DNA vaccines^4–12^. The congenital malformations caused by ZIKV infection during pregnancy has stimulated work on subunit vaccines, as live attenuated and other replicating virus vaccines are contraindicated during pregnancy. Advances in molecular biology and bio/nanotechnology have led to the production of recombinant viral proteins with applications in diagnostics and vaccinology. The flavivirus envelope (E) protein is a major target of neutralizing and protective human antibodies, but recombinantly expressed soluble E protein without the C-terminal transmembrane domains has not proven to be particularly effective as a vaccine antigen^13–16^. For DENV and, more recently ZIKV, we see growing evidence that E protein domains or E proteins expressed as a soluble recombinant antigen (sRecE) fails to induce robust protective responses unlike whole virus or virus-like particle (VLP) vaccine antigens^5, 6, 17–19^. Recent studies have established that complex quaternary structure epitopes displayed by E oligomers on the viral surface but not E monomers are targets of strongly neutralizing and protective human antibodies^6, 7^. In solution, sRecE from flaviviruses are in a dynamic equilibrium that favors the monomer over the dimer, which likely explains the poor binding of strongly neutralizing quaternary epitope directed human antibodies and the overall poor immunogenicity in pre-clinical studies^6, 20, 21^.

**Figure 1:**
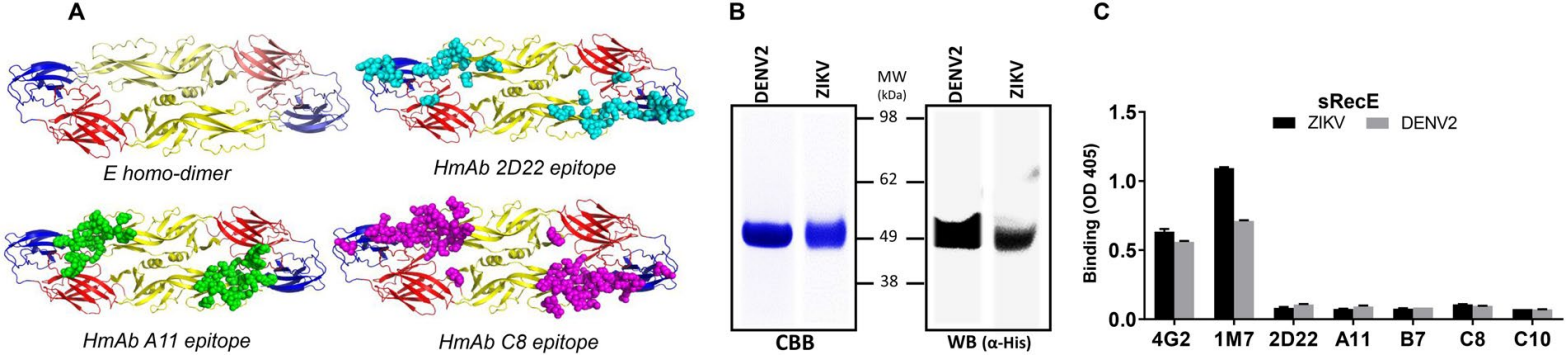
DENV2 and ZIKV sRecE expression and characterization. (**A**) The flavivirus E protein consists of three beta-barrel domains designated domains I (red), II (yellow) and III (blue), with the native protein forming a head-to-tail homo-dimer. The quaternary epitopes recognized by human dimer-dependent Mabs 2D22 (cyan), A11 (green) and C8 (magenta) are indicated^22, 35^. (**B**) DENV and ZIKV sRecE expression was analyzed by Western Blot (anti-His mAbs), CBB (**C**) and by ELISA using 4G2, 1M7, 2D22, A11, B7 (EDE2), C8 and C10 (EDE1) mAbs.

From a patient who had recovered from a primary DENV serotype 2 infection, we previously isolated and characterized a DENV2 serotype-specific strongly neutralizing mAb, 2D22, that binds to a E protein dimer-dependent quaternary epitope (Fig. 1A)^22–24^. Human mAb 2D22 does not bind to the DENV2 sRecE monomer^24^. Additionally, analysis of B-cells from people exposed to repeated DENV infections has led to the discovery of mAbs that target envelope dimer epitopes (EDE) that are conserved between the 4 DENV serotypes and ZIKV^25, 26^. EDE mAbs (Fig. 1A), which cross-neutralize different DENV serotypes and ZIKV to varying degrees, have been divided into two groups (EDE1 and EDE2), based on their binding footprint and sensitivity to the presence or absence of an N-linked glycan in domain I (EDI) of E protein^25^.

In this study we describe novel methods based on the immobilization of sRecE on a matrix or the use dimer-specific human mAbs to promote the temperature dependent assembly and stabilization of sRecE dimers displaying quaternary structure antibody epitopes targeted by human antibodies. The ability to assemble E homo dimers displaying quaternary structure antibody epitopes is an important advance with applications in flavivirus vaccine development and diagnostics.

## Results

### Expression of DENV2 and ZIKV recombinant E-proteins

The ectodomains of the DENV2 and ZIKV E-proteins were expressed using the EXPI293 mammalian transient expression system, containing their native prM sequences, an N-terminal IL2 secretion leader peptide and a C-terminal 6xHis tail for Ni-affinity purification of the recombinant protein. The expression and purity of sRecE monomers from both DENV2 and ZIKV was analyzed by SDS-PAGE and western blot using anti-His mabs (Fig. 1B). Both Coomassie Brilliant Blue staining and western blotting show the presence of pure proteins with the predicted molecular mass for DENV2 (~48 kDa) and ZIKV sRecE (~47 kDa). The purified proteins were subsequently analyzed by ELISA using a panel of mAbs (Table 1) that recognize epitopes on the monomer (4G2 and 1M7) or homo-dimer (2D22, A11, B7, C8 and C10) (Fig. 1A,C). Binding was observed for mAbs 4G2 and 1M7 but not for mAb 2D22, and the EDE mAbs confirming that the monomer was more abundant than the dimer at equilibrium.

**Table 1:** Binding characteristics of used mAbs.

Mab	M/H	Binding	Neutralization *(W/M/S)*	Binding region	Binding DENV serotypes					Ref.
					*DV1*	*DV2*	*DV3*	*DV4*	ZIKV	
*4G2*	M	F-CR	W	DII FL	++	++	+++	+++	+++	41
*2D22*	H	DV2	DV2:S ZIKV:W	DI/DII Q	−	++	−	−	−	22, 36
*1M7*	H	F-CR	M	DII FL	+++	++	+++	+++	+++	27
*A11*	H	F-CR	DV:S ZIKV:W	DI/DII/DII Q	+++	+++	+++	+++	+	38
*B7*	H	F-CR	DV:S ZIKV:W	DI/DII/DII Q	+++	+++	+++	+++	+	38
*C8*	H	F-CR	DV:S ZIKV:S	DI/DII/DII Q	+++	+++	+++	+++	++	38
*C10*	H	F-CR	DV:S ZIKV:S	DI/DII/DII Q	+++	+++	+++	+++	++	38

Characteristics of the mAbs used for dimer assembly. Several mouse or human (M/H) derived mAbs were used for sRecE dimer assembly, indicating their DENV and ZIKV binding potential. Flavivirus cross reactive (F-CR), weakly, medium or strong (W/M/S) neutralizing, E-domain I, II, III (DI, DII, DIII), fusion loop (FL), quaternary epitope (Q).

### Assembly of sRecE dimers and generation of DENV2 specific quaternary epitopes

**Figure 2:**
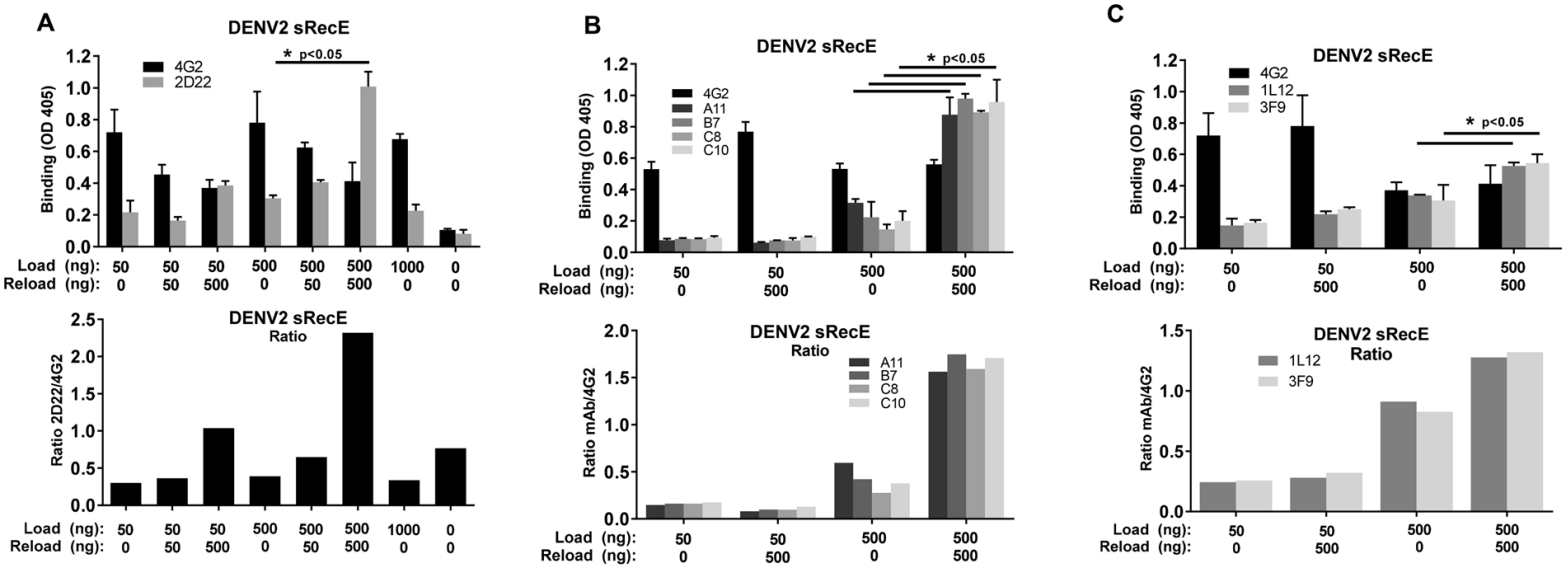
DENV2 sRecE dimer assembly on Ni^2+^-coated surfaces. (**A**) Indicated amounts of DENV2 sRecE were loaded and reloaded on Ni^2+^-coated plates and analyzed for monomeric (4G2) or dimeric protein structures (2D22). Increased dimer formation is displayed as the 2D22/4G2 signal ratio. (**B**) The same assay was performed with EDE1 (A11, B7), EDE2 (C8, C10) and **C)** 3F9 and 1M7 mAbs. No antibody signals were detected when no protein was loaded or reloaded and groups 500 + 0 and 500 + 500 were statistically compared by student T-test (p < 0.05).

As the DENV sRecE antigen does not form stable homo-dimers in solution, we used the C-terminal 6X His tail to immobilize the protein to a Ni^2+^-coated surface under different conditions to promote dimerization. The Ni^2+^-coated surfaces were loaded with different amounts of DENV2 sRecE (50, 500 or 1000 ng/well), blocked and then reloaded with 0, 50 or 500 ng/well of sRecE (Fig. 2A). Dimer formation was assessed by calculation the ratio of dimeric E protein (mAb 2D22 signal) to total E protein (mAb 4G2 signal). Neither mAb bound non-specifically to the Ni^2+^ surface in the absence of antigen (Fig. 2A). Initial loading with 50 or 500 ng of antigen without reloading resulted in a low ratio of 2D22/4G2 binding, indicating the protein was captured and mainly retained as a monomer. When the plates were reloaded with 500 ng of sRecE, we observed a four to seven fold increase in the 2D22/4G2 ratio (Fig. 2A). Augmented 2D22 binding is not a mere antigen concentration effect, since a 1000 ng sRecE load without reload did not show an increased 2D22 signal, whereas a load of 500 ng followed by a reload of 500 ng led to a large increase in 2D22 binding (Fig. 2A). In addition to 2D22, EDE mAbs were used to measure dimer formation of DENV2 sRecE (Fig. 2B). All EDE1 and EDE2 mAbs tested bound well to the 500 ng load/500 ng reload group compared to the other groups confirming the formation of dimers (Fig. 2B **)**. This dimer-building platform was also used to analyze the binding of DENV2 serotype-specific and strongly neutralizing human mAbs 1L12 and 3F9, which bind to complex epitopes that have not been fully mapped yet (Fig. 2C)^27^. Under conditions that favor DENV2 sRecE dimer formation, we observed increased binding of both 1L12 and 3F9.

### sRecE-dimer formation is a temperature dependent process

**Figure 3:**
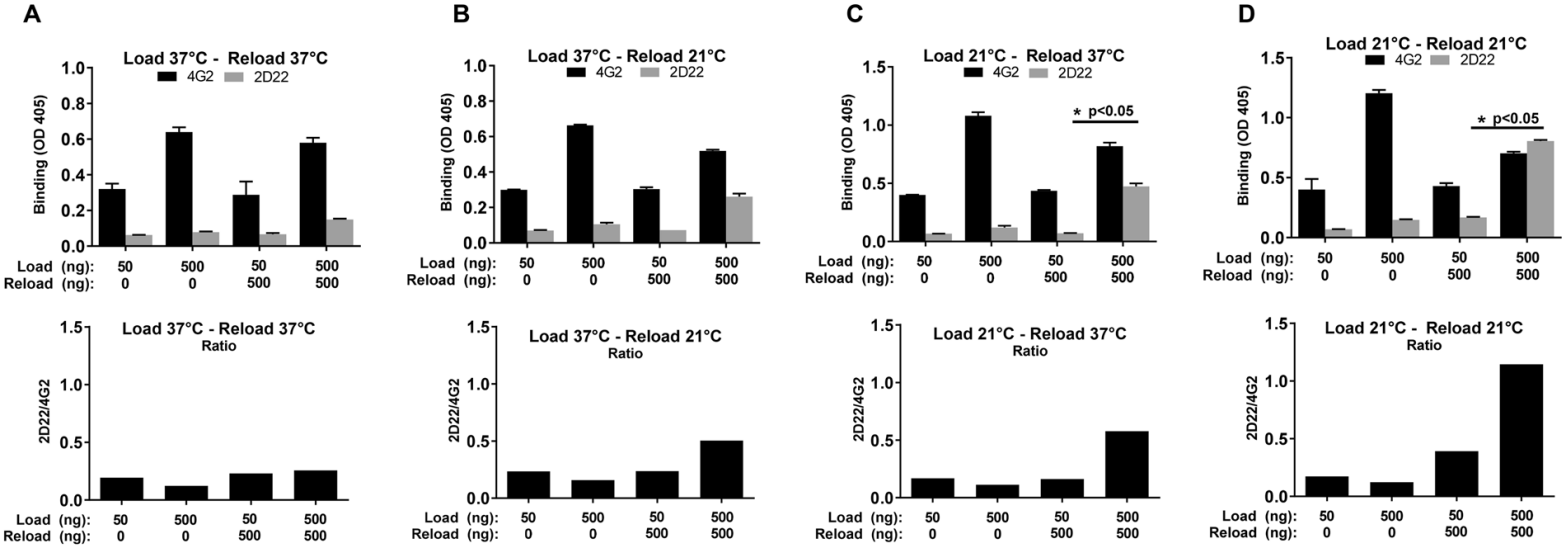
Temperature effects on sRecE dimer formation. Indicated amounts of DENV2 sRecE were loaded and reloaded at different temperatures, resulting in the following temperature regiments: (**A**) Load at 37 °C and reload at 37 °C, (**B**) load at 37 °C and reload at 21 °C, (**C**) load at 21 °C and reload at 37 °C and (**D**) load at 21 °C and reload at 21 °C. Dimer formation was analyzed by 4G2 and 2D22 binding. Groups 500 + 0 and 500 + 500 were statistically compared by student T-test (p < 0.05).

To assess the effect of temperature on E dimer formation on Ni^2+^ plates, DENV2 sRecE was immobilized at 37 °C or 21 °C. After blocking, the wells were reloaded at 37 °C or 21 °C and analyzed with 4G2 and 2D22. A decrease in dimer formation was observed when sRecE was incubated at 37 °C during the immobilization and reloading step (Fig. 3A). Reloading the protein at 21 °C resulted in a modest increase in dimerization (Fig. 3B). A similar increase in the 4G2/2D22 ratio was observed when sRecE was immobilized at 21 °C and reloaded at 37 °C (Fig. 3C). Dimer formation was most efficient when the immobilization and reloading steps were conducted at 21 °C (Fig. 3D), indicating a strong temperature dependency towards the formation of dimer dependent epitopes in this assay.

### Antibody mediated dimer stabilization

**Figure 4:**
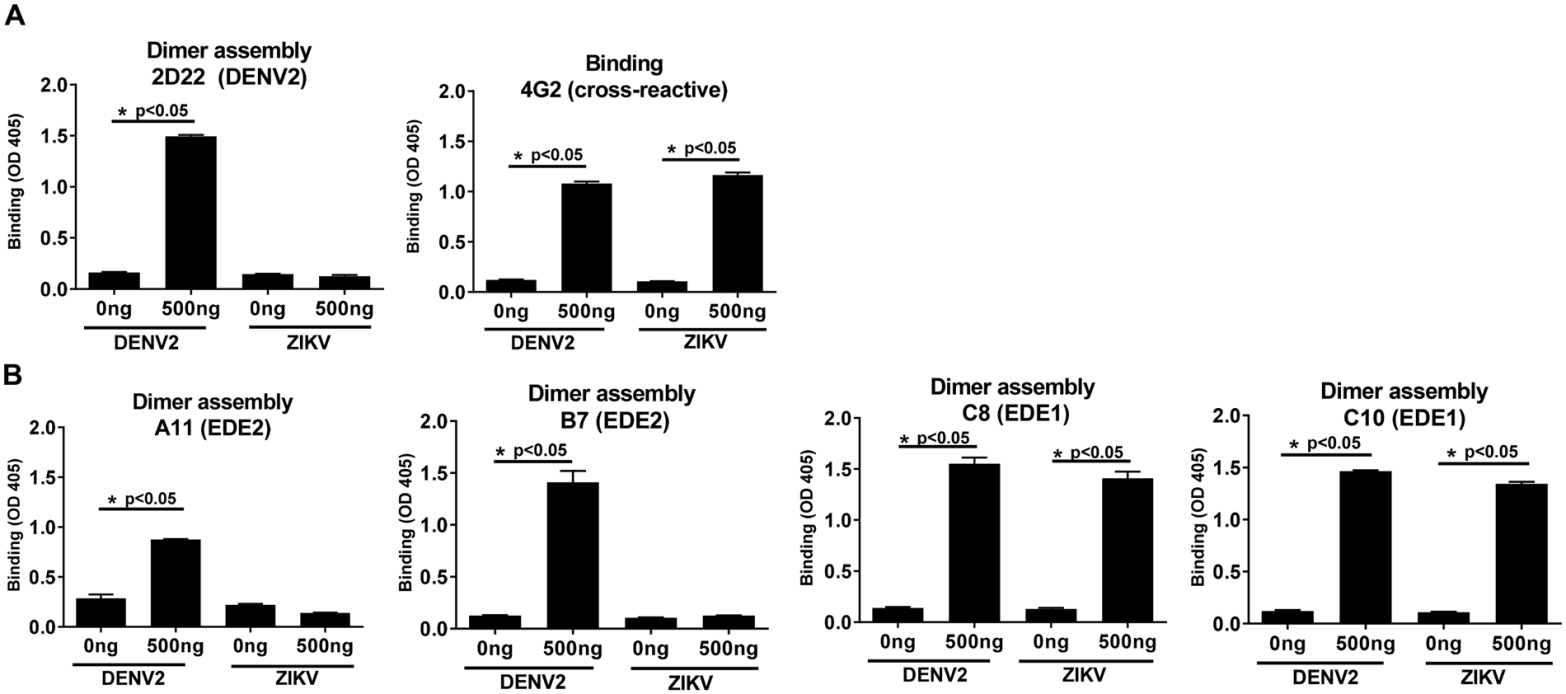
Antibody mediated dimer assembly. Indicated amounts of DENV and ZIKV sRecE were incubated with 500 ng of (**A**) 2D22, 4G2, (**B**) A11, B7, C8 and C10 mAbs in solution. Protein/mAb complexes were loaded on Ni^2+^-coated plates and captured antibodies were detected. Statistical analysis was performed using the student T-test.

In solution sRecE is likely to be present as both monomers and dimers with the equilibrium favoring the monomers under our experimental conditions. As an alternative approach to producing stable E protein dimers, we incubated sRecE in solution with dimer-dependent mAbs. sRecE (500 ng) was incubated with 500 ng of mAbs 2D22, A11 and B7 (EDE2), and C8 and C10 (EDE1). sRecE/mAb complexes were subsequently captured through His tag at the C terminal of sRecE to Ni^2+^-coated ELISA plate. Any mAb bound to the captured sRecE was detected using a secondary goat anti-human IgG-AP conjugated antibody. When 2D22 or 4G2 were incubated without DENV or ZIKV sRecE subunits (0 ng/well), no mAb-protein complex was captured indicating that the mAbs did not directly bind to Ni2+ plates (Fig. 4A). A large increase in the detection of mAb 2D22 was observed after incubation with DENV2 sRecE, and, as expected, 2D22 failed to bind ZIKV sRecE **(**Fig. 4A **)**. Similar binding patterns were obtained after EDE mAbs were mixed with DENV2 sRecE proteins in solution (Fig. 4B). These results indicate that dimer dependent human mAbs can stabilize DENV2 E dimers in solution and preserve the dimers during capture to Ni^2+^-plates. Interestingly for ZIKV sRecE, only EDE1 mAbs C8 and C10 were detected, while EDE2 antibodies did not bind.

**Figure 5:**
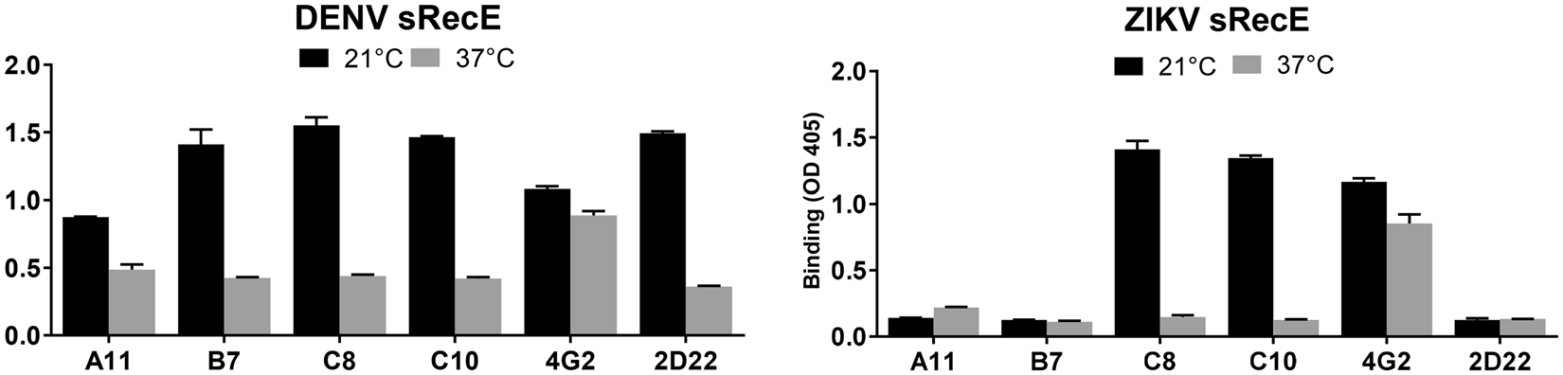
Antibody mediated dimer assembly is a temperature dependent process. The effect of temperature on mAb mediated dimer assembly was analyzed by incubating 500 ng mAb with 500 ng DENV2 or ZIKV sRecE. sRecE-mAb incubations were performed at 21 °C or 37 °C and loaded on Ni^2+^-coated plates and captured antibodies were detected.

The binding assay was repeated by incubating the mAbs and sRecE proteins at 21 °C and 37 °C to determine the impact of temperature on dimer formation and mAb mediated stabilization in solution (Fig. 5). The dimer dependent mAbs stabilized both DENV2 and ZIKV E dimers poorly at 37 °C compared to 21 °C. Similar to the assembly of sRecE dimers on Ni^2+^-surfaces, these results indicate a strong temperature dependence on the formation and mAb mediated stabilization of E dimers.

## Discussion

Recent studies show that flavivirus virions or virus-like particles (VLPs) are better vaccine antigens than sRecE^5, 6^. The poor immunogenicity of sRecE may be, in part, due to the failure to efficiently display E protein quaternary structure epitopes displayed on virions and VLPs that are known to be targets of strongly neutralizing human mAbs. In this study, we present novel methods to assemble or stabilize dimer-dependent quaternary epitopes out of monomeric sRecE building blocks.

**Figure 6:**
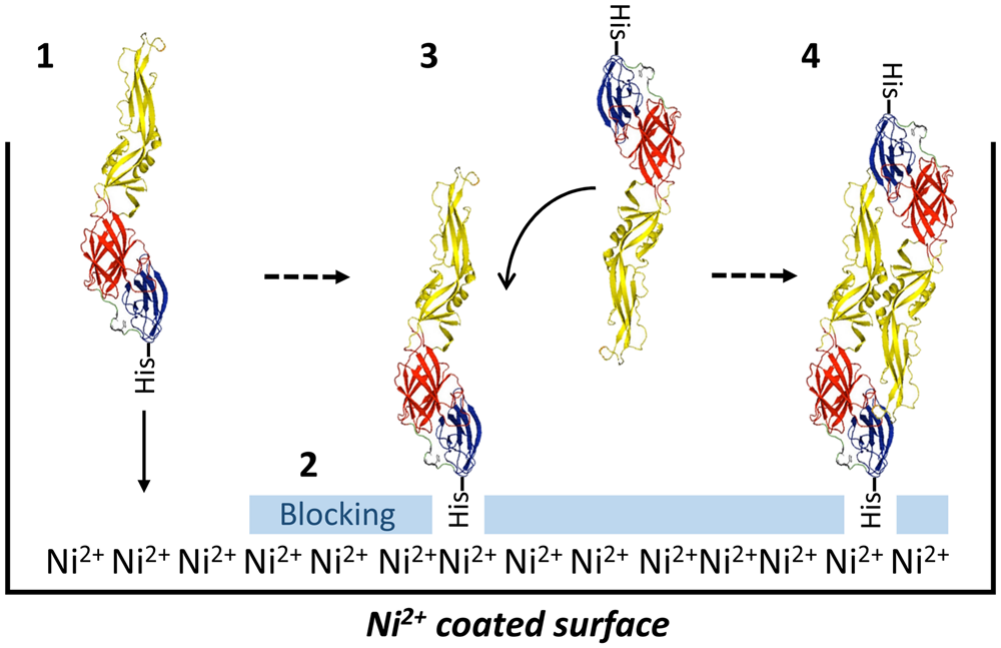
Schematic representation of the postulated model for sRecE-dimer assembly from immobilized monomers. (**1**) sRecE is chelated to Ni^2+^-coated plates. The immobilization of sRecE at its C-terminal end presumably locks the protein in a specific conformation. (**2**) The plates are subsequently blocked and (**3**) reloaded with sRecE at high protein concentrations. (**4**) This enables interaction of the immobilized sRecE with the reloaded proteins and generates quaternary epitopes that can be recognized by E-dimer epitope dependent mAbs.

The binding of 2D22 and the EDE mAbs to reloaded wells demonstrates the formation of E homo-dimers displaying quaternary epitopes, that were absent in the non-reloaded wells. During the initial sRecE load, proteins are chelated to the Ni^2+^-surface through their C-terminal His-tag. This interaction is anticipated to immobilize the proteins in a confirmation that enables interaction and dimer formation with the reloaded sRecE (Fig. 6). In contrast to the homo-dimers, the dimer-dependent binding mAbs might bind the partial epitope present on monomers with no or very low binding affinity. The C-terminal attachment of the proteins to the Ni2+-surface replicates the native C-terminal anchoring of E-proteins to lipid membranes. To transfer this technology for vaccine purposes, further platform development is required where the Ni^2+^-His immobilization is replaced by more biological compounds suitable for human administration.

DENV virion morphology is affected by several factors such as the binding of mAbs, pH and the proteolytic processing of envelope proteins^28–30^. In addition, some DENV strains have been shown to undergo temperature dependent conformational changes in their envelope structure. At lower temperatures, the DENV2 E protein lies flat on the virus surface in a compact manner, resulting in a smooth appearance. At 37 °C however, E protein becomes less compact and undergoes conformational changes leading to a ‘bumpy’ surface^31–33^. Similar effects on proteins structure and organization have recently been seen in other sRecE expression studies^34^. The effect of temperature in the assembly of DENV2 and ZIKV sRecE dimers on artificial surfaces is apparent. Temperature might affect the flexibility of sRecE to be able to pair with the reloaded or immobilized counterpart. In this case, the actual number of dimers will be affected. Another explanation would be that at both temperatures dimers are formed with equal efficiency, but at the higher temperature the 2D22 epitope may be disrupted or hidden.

The sRecE proteins are expressed in an equilibrium between dimers and monomers, with an emphasis towards the monomeric state. Using static light scattering after size exclusion chromatography, we confirmed that the majority of sRecE was monomeric in solution and that the addition of dimer dependent mAbs in solution stabilized E dimer/mAb complexes (Fig. S1). For DENV2 sRecE dimers, all tested mAbs stabilized dimers prior to capture on Ni^2+^-plates. For ZIKV sRecE however, only EDE1 mAbs were able to stabilize dimers, whereas EDE2 mAbs failed to do so. The ability of EDE1 mAbs to stabilize ZIKV sRecE-dimers is consistent with previous work showing better binding and neutralization of ZIKV by EDE1 compared to EDE2 mAbs^35–37^. For DENV, all EDE mAbs occupy two binding sites within one E-dimer^38^. In case of ZIKV however, EDEs dock in different angels compared to DENV. Where EDE1 still occupies two binding sites, EDE2 A11 only occupies one site. Docking of a second Fab is most likely inhibited by steric hindrance by proximate complexes^35^. This might be a reason why the low affinity binding EDE2 mAbs are not able to stablize ZIKV sRecE dimers in solution.

The magnitude, explosive spread and severe clinical manifestations of DENV and ZIKV epidemics demand research towards reliable diagnostic tools and vaccines. Considering that pregnant women could be a potential target group for ZIKV vaccines, safe alternatives to live attenuated and replicating virus vaccines, such as subunit approaches, are in urgent demand. More broadly, investigators are focusing on methods to produce recombinant antigens that display complex quaternary epitopes on the viral surface targeted by the human immune response^39, 40^. Here we demonstrate the feasibility of assembling flavivirus E protein structures that display complex quaternary epitopes recognized by both virus specific and cross-reactive, broadly neutralizing antibodies. Flavivirus sRecE protein dimers hold much promise as antigens for advancing diagnostics and subunit vaccines.

## Methods

### Expression and purification of DENV2 and ZIKV sRecE

The soluble recombinant DENV2 (sRecE, aa 1–395) and ZIKV (aa 1–404) envelope proteins were expressed by the EXPI293 transient expression system (ThermoFisher) as previously described^24^. In short: DENV2 and ZIKV sRecE was equipped with a C-terminal 6x His tag and expression was driven by a CAG promoter. Through tangential flow filtration, supernatants were concentrated and buffer exchanged into a Ni^2+^ binding buffer (50 mM NaPO_4_, 500 mM NaCl, 25 mM imidazole, 0.02% Na-Azide) and subsequently Ni^2+^ affinity purified. After washing, the Ni^2+^ column was serially eluted with elution buffer (50 mM NaPO_4_, 500 mM NaCl, 500 mM Imidazole 0.02%, Na-Azide, 10% glycerol). Fractions containing envelope proteins were pooled subjected to size exclusion chromatography using a 16/60 Superdex S200^TM^ column. Fractions with envelope protein were pooled in PBS + 10% glycerol, concentrated and flash frozen in liquid N_2_, and stored at −80 °C. The oligomeric state of the purified proteins were analyzed size exclusion chromatography using a Superdex S200^TM^ column connected to multi angle light scattering instrument (Wyatt DAWN HELEOS-II) with an OptiLab T-rex refractometer. 200 µg of DENV2 sRecE was analyzed with or without 40 µg of 2D22 in 100 µl PBS.

### Protein analysis by Western blot and CBB stain

DENV2 and ZIKV sRecE expression was analyzed by Coomassie Brilliant Blue (CBB) staining and Western blot. Protein samples were added to Laemmli sample buffer containing 20 mM ß-mercaptoethanol. Samples were boiled for 3 mins and loaded on a NuPAGE^TM^, NOVEX^TM^ 4–12% Bis-Tris protein gel (Invitrogen). Proteins were transferred to a polyvinylidene difluoride (PDVF) membrane and blocked for 1 hr with 0.5% skim milk in PBS + 0.1% Tween 20 (PBST). Next, membranes were incubated with anti-His-HRP conjugated antibody (Invitrogen) 1:5000 diluted in PBST for 1 hr. Membranes were washed 3 times with PBST and developed with Amersham ECL Prime Wester Blotting Detection Reagent (GE Healthcare).

### Protein analysis by ELISA

100 ng/well (in TBS) of purified DENV2 or ZIKV sRecE was loaded on Ni^2+^-coated wells (Pierce) for 1 hr at RT. Next, wells were blocked with 3% skim milk in TBS + 0.05% Tween-20 (TBST) for 1 hr at RT and after washing with TBST, wells were incubated with 100 ng/well of 4G2, 1M7, 2D22, EDE1 (C8, C10) or EDE2 (A11, B7) mAbs in TBST for 1 hr at RT. Wells were subsequently washed and 4G2 treated wells were incubated with 1:1000 anti-mouse IgG-AP conjugated (Sigma) and 1M7, 2D22, EDE1 and EDE2 treated wells were incubated with 1:2500 anti-human IgG-AP conjugated (Sigma). After washing, plates were developed using AP-substrate (Sigma). Absorbance was measured at 405 nm.

### Quaternary epitope assembly ELISA

To assemble DENV2 sRecE dimers, 50 ng and 500 ng/well of sRecE was loaded on clear Ni^2+^-coated ELISA plates (pre-blocked with BSA, 9 pmol binding capacity, purchased from Pierce, #15142) in 50 µl/well TBS for 1 hr at room temperature (RT, 21 °C). Wells were subsequently blocked with 3% skim milk in TBS + 0.05% Tween-20 (TBST) for 1 hr at RT. Wells were washed in TBST and reloaded with 0 ng, 50 ng or 500 ng/well sRecE and incubated for 1 hr at RT. Next, wells were washed 3 times with TBST and incubated with 100 ng/well of 4G2, 2D22, EDE1 (C8, C10), EDE2 (A11, B7), 1L12 or 3F9 mAbs in blocking buffer for 1 hr at RT. After washing, 4G2 treated wells were incubated with 1:1000 anti-mouse IgG-AP conjugated (Sigma) and 2D22, EDE1, EDE2, 1L12 and 3F9 treated wells were incubated with 1:2500 anti-human IgG-AP conjugated (Sigma). Plates were subsequently washed and developed using AP-substrate (Sigma). Absorbance was measured at 405 nm.

To analyze the effect of temperature on dimer-assembly, Ni^2+^-coated ELISA plates were loaded with 50 ng or 500 ng/well of sRecE in TBS for 1 hr at 37 °C or 21 °C. Plates were blocked with 3% skim milk in TBST for 1 hr at 21 °C or 37 °C. After washing in TBST, wells were reloaded with 0 ng or 500 ng/well sRecE in TBST for 1 hr at 21 °C or 37 °C. This way, the following loading-reloading regiments were created: Load at 37 °C and reload at 37 °C, load at 37 °C and reload at 21 °C, load at 21 °C and reload at 37 °C, load at 21 °C and reload at 21 °C. After washing, plates were incubated with 100 ng/well of 4G2 or 2D22 in blocking buffer for 1 hr at RT. Next, plates were washed and 4G2 treated wells were incubated with 1:1000 anti-mouse IgG-AP conjugated (Sigma), EDE2, 1L12 and 3F9 treated wells were incubated with 1:2500 anti-human IgG-AP conjugated (Sigma). Plates were subsequently washed and developed using AP-substrate (Sigma). Absorbance was measured at 405 nm. The binding ratios (mAb/4G2) were determined by dividing the means of the mAb binding signals.

### Antibody mediated dimer stabilization

0 ng, or 500 ng of sRecE in TBS + Tween-20 (TBST) was incubated with 500 ng of EDE1 (C8, C10), EDE2 (A11, B7), 2D22 and 4G2 mAbs (in TBST) for 1 hr, shaking at RT (21 °C). The sRecE/mAb mix was plated on Ni^2+^-coated plates and incubated for 1 hr at RT. Next, wells were blocked in 3% skim milk in TBS + 0.05% Tween-20 for 1 hr at RT. After washing with TBST, 4G2 treated wells were incubated with 1:1000 anti-mouse IgG-AP conjugated (Sigma) and EDE1, EDE2 and 2D22 treated wells were incubated with 1:2500 anti-human IgG-AP conjugated (Sigma). Following washing, wells were developed using AP-substrate (Sigma). Absorbance was measured at 405 nm. To analyze the effect of temperature on mAb mediated dimer formation, 500 ng of sRecE was incubated with 500 ng of mAb. The previously described ELISA was repeated with incubation steps performed at 21 °C or 37 °C.

### Data availability statement

All data generated or analyzed during this study are included in this published article (and its Supplementary Information files).

## Electronic supplementary material

Supplementary Information
