# Supplementary material for: In Vitro Assembly and Stabilization of Dengue and Zika Virus Envelope Protein Homo-Dimers
Source: Sci Rep. 2017 Jul 3;7:4524. doi: 10.1038/s41598-017-04767-6 (PMC5495877; doi:10.1038/s41598-017-04767-6)
Supplement: Supplementary file 1 — Supplementary Information [file 41598_2017_4767_MOESM1_ESM.pdf]

# Supplementary information.

## *In Vitro* Assembly and Stabilization of Dengue and Zika Virus Envelope Protein Homo-Dimers

Stefan W.Metz[1*], Emily N.Gallichotte[1,3], Alex Brackbill[2], Lakshmanane Premkumar[1], Michael J. Miley[2], Ralph Baric[1,3], Aravinda M. de Silva[1*].

1: Department of Microbiology and Immunology, University of North Carolina School of Medicine, Chapel Hill, NC, USA

2: Department of Pharmacology, University of North Carolina School of Medicine, Chapel Hill, NC, USA

3: Department of Epidemiology, University of North Carolina School of Public Health, Chapel Hill, NC, USA

*: Corresponding authors

Stefan Metz: swmetz@med.unc.edu

Aravinda de Silva: aravinda_desilva@med.unc.edu

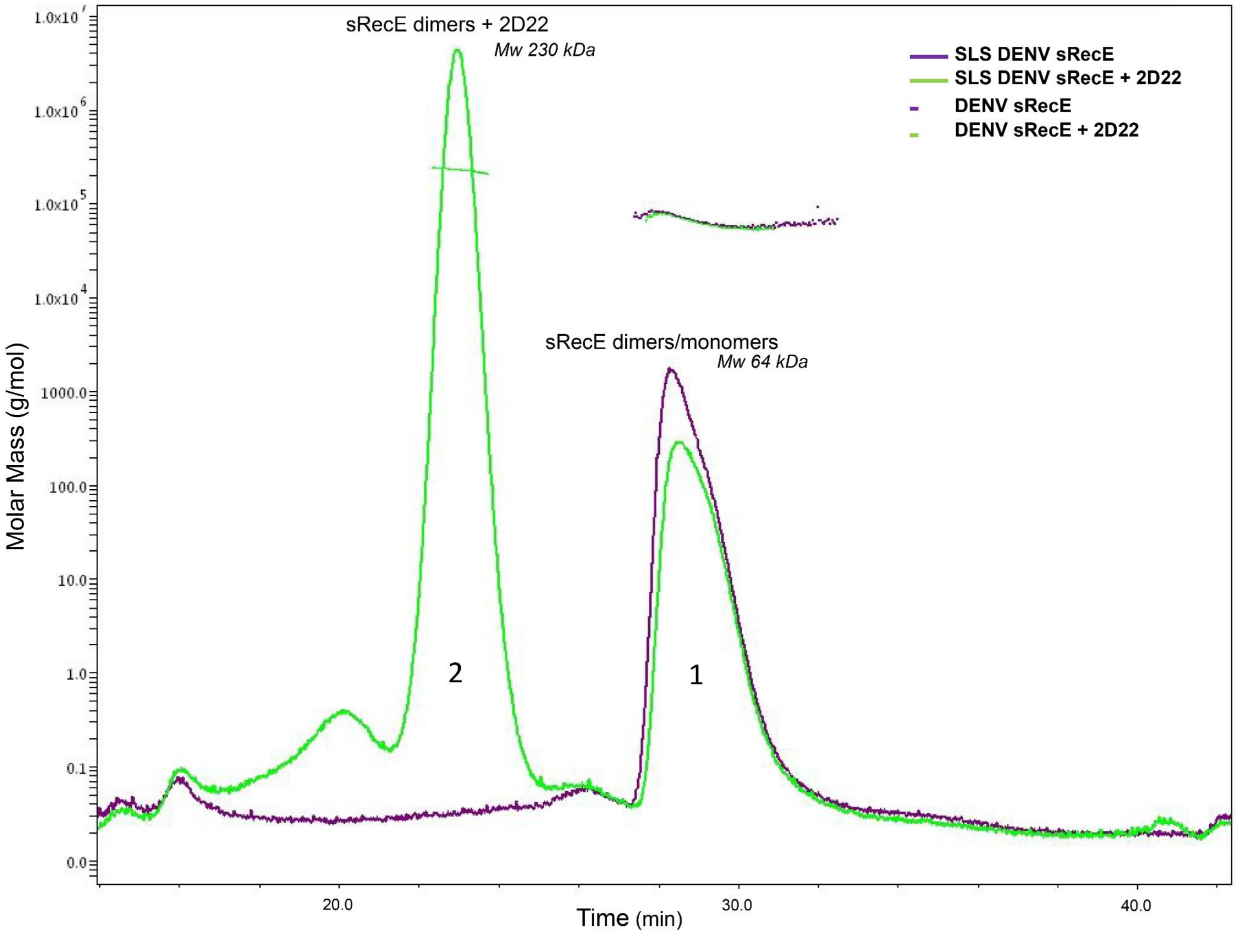


**Supplement Figure 1: DENV sRecE oligomeric state analysis.**

The oligomeric state of 200 µg of DENV2 sRecE with (green) or without (purple line) 40 µg of 2D22 in 100 µl PBS were analyzed by static light scattering (SLS) after size exclusion chromatography. Peak 1 represents the sRecE dimer/monomer equilibrium, with an average calculated molecular weight (Mw) of 64 kDa. The second peak represents sRecE dimers in association with the 2D22 mAb and has a predicted Mw of 230 kDa. The purple (sRecE) and green (sRecE + 2D22) dotted lines represent the calculated molar mass of the protein fractions.
